# Evaluation of AlphaFold modeling for elucidation of nanobody–peptide epitope interactions

**DOI:** 10.1016/j.jbc.2025.110268

**Published:** 2025-05-21

**Authors:** Shivani Sachdev, Swarnali Roy, Shubhra J. Saha, Gengxiang Zhao, Rashmi Kumariya, Brendan A. Creemer, Rui Yin, Brian G. Pierce, Carole A. Bewley, Ross W. Cheloha

**Affiliations:** 1 Laboratory of Bioorganic Chemistry; National Institutes of Diabetes, Digestive, and Kidney Diseases; National Institutes of Health, Bethesda, Maryland, USA; 2 University of Maryland Institute for Bioscience and Biotechnology Research, Rockville, Maryland, USA; 3 Department of Cell Biology and Molecular Genetics, University of Maryland, College Park, Maryland, USA

**Keywords:** nanobody, epitope, peptide, AlphaFold, antibody, single-domain antibody, crosslinking

## Abstract

Models of antibody (Ab)–antigen complexes can be used to understand interaction mechanisms and for improving affinity. This study evaluates the use of the protein structure prediction algorithm AlphaFold (AF) for exploration of interactions between peptide epitope tags and the smallest functional Ab fragments, nanobodies (Nbs). Although past studies of AF for modeling Ab–target (antigen) interactions suggested modest algorithm performance, those were primarily focused on Ab–protein interactions, while the performance and utility of AF for Nb–peptide interactions, which are generally less complex because of smaller antigens, smaller binding domains, and fewer chains, is less clear. In this study, we evaluated the performance of AF for predicting the structures of Nbs bound to experimentally validated, linear, short peptide epitopes (Nb–tag pairs). We expanded the pool of experimental data available for comparison through crystallization and structural determination of a previously reported Nb–tag complex (Nb_127_). Models of Nb–tag pair structures generated from AF were variable with respect to consistency with experimental data, with good performance in just over half (four of six) of cases. Even among Nb–tag pairs successfully modeled in isolation, efforts to translate modeling to more complex contexts failed, suggesting an underappreciated role of the size and complexity of inputs in AF modeling success. Finally, the model of an Nb–tag pair with minimal previous characterization was used to guide the design of a peptide–electrophile conjugate that undergoes covalent crosslinking with Nb upon binding. These findings highlight the utility of minimized Ab and antigen structures to maximize insights from AF modeling.

Decades of intensive study have provided a rich collection of data that link protein primary structure (sequence) with structure (1). In parallel, impressive advances in technologies for DNA sequencing have generated a compendium of sequence information covering a large swath of organisms alive today (2). Modern computational methodology has leveraged rich libraries of DNA sequence information, evolutionary analysis, and structural databases to generate algorithms for predicting structures of previously unanalyzed or fully novel proteins (3, 4). Protein structure prediction methodology has realized substantial improvements in speed and performance following the publication and widespread implementation of the AlphaFold2 (AF2) algorithm (5). Refinements of this methodology have enabled high-quality predictions of the structures of proteins involved in protein–protein interactions (PPIs) (6). One important class of PPIs that has often been poorly modeled *via* computation is the interaction between antibodies (Abs) and their targets (antigens) (7). Notably, AF2 performed better than other comparable modeling methods, although there remains room for improvement (8, 9). Further improvement in these efforts has been realized with the release of AlphaFold3 (AF3) (10). This work focuses on application of AF2 given its widespread characterization and the abundance of tools available for easy implementation.

Ab–antigen interactions represent a challenge for prediction algorithms that use coevolution of amino acid residues for predicting protein folding and PPI interfaces, such as AF2 (11). Genetic recombination, somatic (hyper)mutation, and clonal selection drive sequence diversification of Abs produced by the immune system over a time frame that is short by human evolutionary standards. For example, a single immunization or infection in humans can result in generation of many novel Ab sequences that bind to antigens through new or unusual mechanisms (12). This complex process of immune system–mediated Ab sequence variation, typically without a change in antigen sequence, and its impact on binding to antigens of interest, is incompletely captured in sequence data used to train modeling algorithms. Further difficulty in modeling arises from the mechanism through which Abs bind to their targets. The binding surface for Abs occurs primarily over six complementarity-determining regions (CDRs), loops that are evenly distributed between the Ab heavy chain (HC) and light chain (Fig. 1*A*) (13). Simplified systems to assess modeling Ab–antigen interactions could be useful for guiding improvements in modeling approaches.

**Figure 1:**
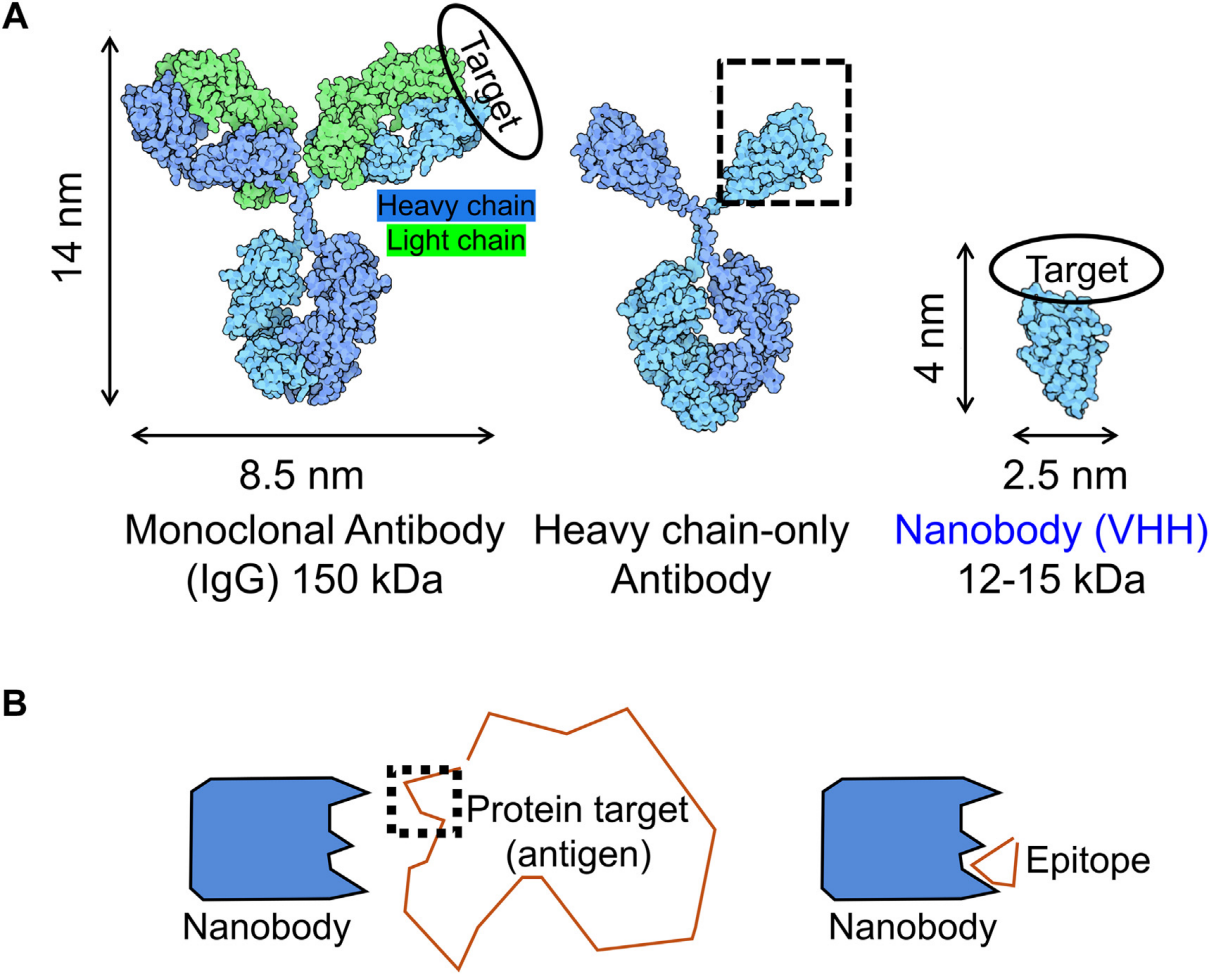
**Illustration of antibody (Ab) and antigen complexity.***A,* comparison of full-sized Abs to heavy chain–only Abs and nanobodies (Nbs). *B,* comparison of full-sized protein antigens to epitope fragments.

Unlike conventional Abs from humans and mice (immunoglublins G [IgGs]), which rely on an Ab structure comprised of HC and light chain polypeptides, camelids (camels, alpacas, and llamas) also produce a type of Ab made only from HC (14). Although the physiological role of HC-only Abs is not clear, the single-chain architecture has proven valuable for many biological applications (15). Excision of the antigen-binding domain of HC-only Abs provides variable HC domains of HC-only Abs (VHHs, also known as nanobodies [Nbs], Fig. 1*A*). Nbs represent the smallest Ab fragment that maintains high antigen-binding affinity. Nbs are 10% the size of conventional IgGs, and unlike IgGs, they frequently do not require disulfide bonds or glycosylation for folding or function (16). Because of these features, Nbs can be recombinantly expressed in bacteria in high yield. Nbs have been widely used to stabilize conformationally flexible proteins for structural studies and imaging applications (17, 18). Site-specific labeling of Nbs has been achieved using a variety of chemical and enzymatic approaches, which has facilitated the production of Nb–ligand conjugates for the study of cell surface receptor signaling (19, 20, 21, 22). Features of Nbs potentially useful for modeling applications are their small size, rigid single-domain architecture, and binding mechanism that relies on only three CDRs (*versus* six for conventional IgGs). Indeed, a higher level of success was observed in modeling of Nb–antigen complexes compared with Ab–antigen complexes (8).

Antigen size and complexity constitute other variables that are potentially important to the success of modeling Ab–antigen interactions. Although many Abs and Nbs bind to a discontinuous (conformational) epitope on their target, a substantial fraction bind primarily through a continuous stretch of amino acids (linear epitope). For some linear epitopes, these sequences can be extracted and synthesized chemically or fused to a partner protein for evaluation of binding outside the context of the natural protein antigen. This approach is often used in epitope tagging, wherein target proteins for which high-quality Ab detection reagents are unavailable, are modified to contain an epitope tag recognized by a high-quality Ab (23). Evaluation of Abs or Nbs that bind short linear epitopes offers the possibility of comparing the modeling output generated for Ab–full size antigen complexes to those of Ab–epitope complexes (Fig. 1*B*). In this work, we analyze previously identified Nb–linear peptide epitope (tag) interactions (24, 25, 26, 27, 28, 29, 30, 31, 32) through AF2 modeling. We restrict our analysis to Nb–peptide epitope pairs (six total) that meet one of these criteria: experimental structural information is available, substantial structure–activity relationship studies have been performed, or our group is actively involved in their characterization. By restricting our analysis to Nbs that bind to small peptide epitopes, we highlight the important role of epitope complexity in modeling success and the unpredictable nature of modeling epitope recognition in different protein contexts. We further expand this line of inquiry by characterizing for the first time the structure of an Nb–tag complex *via* X-ray crystallographic analysis. Furthermore, we demonstrate that AF can be used to predict the binding of Nbs to variants of peptide epitopes, which results in the identification of analogs with improved Nb affinity. Finally, we show that AF modeling can be used to guide the design of epitope peptide–electrophile conjugates that form a covalent bond with target upon Nb binding (33).

## Results

**Figure 2:**
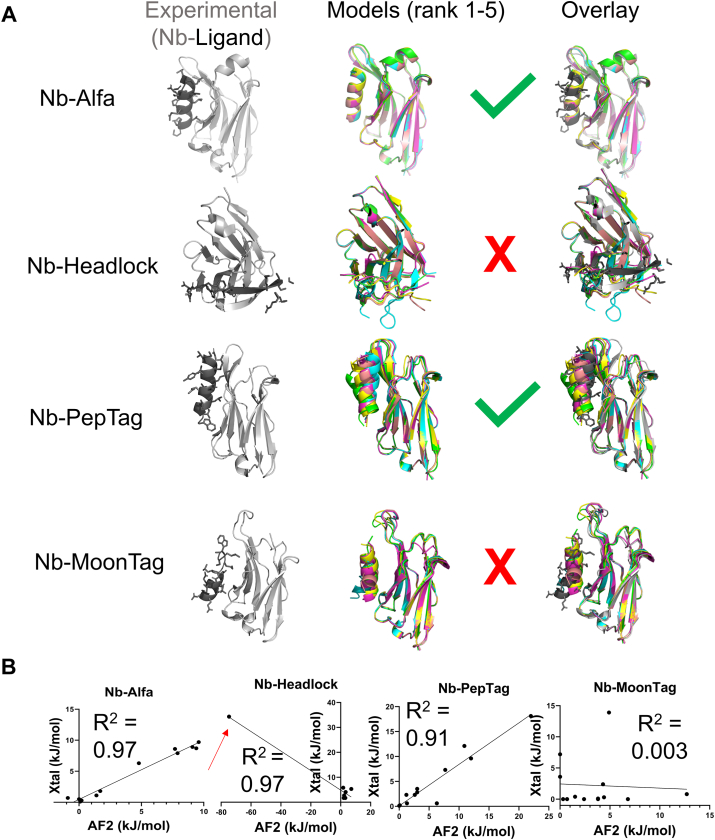
**Comparison of experimental Nb–epitope structures to AF2-generated models.***A,* experimental structures (*gray* and *black*, *left*) are compared with an overlay of five complex models produced by AF2 (*middle*, color) *via* overlay (*right*). Structures and overlays were produced using PyMOL. *B,* quantitative assessment of peptide epitope side-chain energetic contributions to binding. The energetic contribution of each side chain within experimental or the top ranked AF2-generated complex was quantified using BUDE Ala Scan. Each side chain was plotted as a single point in a scatter plot. A linear correlation model was used to generate a trendline in GraphPad Prism. A *red arrow* highlights a residue that forms a steric clash in an AF2-generated model. AF2, AlphaFold2; Nb, nanobody.

**Figure 3:**
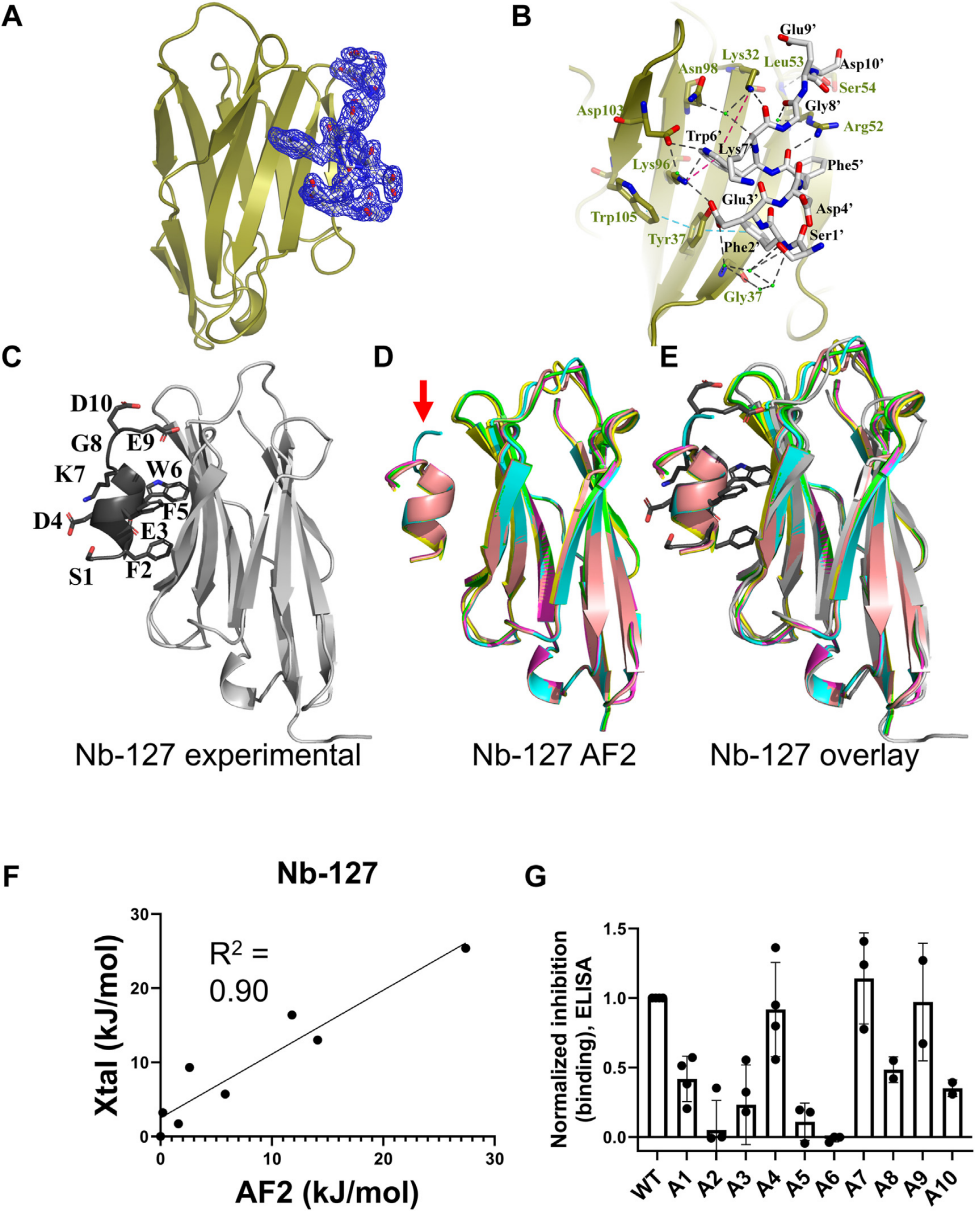
**Comparison of a new Nb_127_–eptiope peptide complex structure with AF2-generated models.** A complex of Nb_127_ and 127-tag was crystallized and analyzed as described in the Experimental procedures section. *A,* depiction of peptide epitope electron density. The Nb is shown in *cartoon* and the 127-tag in *sticks* with σ = 1 map in *blue mesh*. *B,* detailed map of Nb_127_–peptide interaction. 127-tag is shown in *sticks* (*black text for residue labels*) and Nb in *cartoon*, with residues of Nb interacting with 127-tag in sticks with *goldtext labels*. *Black dashes* show hydrogen bonding interaction within the complex. The *cyan dashed line* shows a pi–pi contact between Trp105, Tyr37, and Phe2′ (4.7 Å and 5.0 Å, respectively). The *red dashed line* shows the Pi–cation contact between Lys32, Lys96, and Trp6′ (3.3 Å and 6.5 Å, respectively). *Green balls* show waters involved in intermediating the H-bond between Nb and 127-tag. *C*–*E,* an experimental structure (*C*) is compared with an overlay of five complex models produced by AF2 (*D*) *via* overlay (*E*). *F,* quantitative assessment of peptide epitope side-chain energetic contributions to binding, performed as described in Figure 2 and Experimental procedures section. *G,* experimental evaluation of the binding of 127-tag variants to Nb_127_ using ELISA. Peptide characterization is shown in Table S5. Higher bars correspond to more complete inhibition and stronger binding (see the Experimental procedures section). Data correspond to mean ± SD from three or more independent replicates. AF2, AlphaFold2; Nb, nanobody.

We performed a survey of the literature to compile a collection of Nbs that bind to short (<20 residue), linear peptide epitopes. We focused on Nb–peptide interactions for which structural information was available (Fig. 2) or those used previously for in-house studies (Fig. 3, Table S1 (24, 25, 26, 27, 28, 29, 30, 31, 32)). We compared four structurally characterized Nb–peptide complexes to structural models generated using AF2 multimer version 3 in ColabFold (34). Unless otherwise indicated, all models were generated using this implementation of ColabFold. Of the four experimental Nb–tag structures, all were deposited into online repositories prior to date (September 30, 2021) at which the AF2 multimer version 3 training set was extracted (Table S1). We used AF2 to generate an ensemble of five structural models for each of these Nb–peptide tag complexes. We superimposed the ensemble (Fig. 2*A*, *middle*) and the experimental structure (Fig. 2*A*, *right*) using PyMOL. Visual inspection shows a substantial variation in consistency for both the ensemble and model-experimental alignments. For the Nb_Alfa_–tag interaction, there was a close alignment between all the modeled complexes and the experimentally characterized complex (Fig. 2*A*) (28). Nb_PepTag_ models were more variable, with one model (rank 1, *cyan*) closely resembling the experimental structure and others showing more divergence (Fig. 2*A*). For Nb_headlock_ and Nb_MoonTag_, there were notable differences between the experimentally determined complex structures (26) and the structures generated by modeling (Fig. 2*A*). Past work (7, 8) showed that confidence parameters generated by AF offer predictive power for assessing protein–protein complex model accuracy. We observe an analogous trend here (Table S2). All ColabFold models with interface predicted template modeling (ipTM) scores above 0.8 (all Nb_Alfa_ models and the top ranked Nb_PepTag_ model) showed good agreement with experimental structures, with interface and peptide RMSD values below 2 Å and medium or high accuracy based on Critical Assessment of Predicted Interactions (CAPRI) criteria (35). In contrast, complexes with lower ipTM scores showed lower accuracy, measured by RMSD and by visual inspection of overlayed models (Fig. 2*A*).

These complexes were assessed through analysis of the Nb–peptide interface *via* computational alanine scan (Fig. 2*B*) (36, 37). The consequences of replacing each (non-Ala) residue within the peptide epitope with Ala were quantified as a computationally derived ΔΔG value. These computational ΔΔG values were compared between experimental structures and the top ranked AF models for each position within peptide epitopes. If the peptide–Nb interface generated in the AF model closely resembles the experimental interface, there should be a positive and roughly linear correlation when plotting ΔΔG-experimental *versus* ΔΔG-model. Analysis of the Nb_Alfa_– and Nb_PepTag_–peptide complexes using this approach yields a positive and linear correlation, whereas other complexes show either a negative correlation, poor concordance between experimental and modeling structures, or both. This analysis shows that the AF2-generated model of the Nb_headlock_ complex places an amino acid residue such that replacement with Ala is predicted to improve binding, suggesting the presence of a steric clash in the AF2 models (Fig. 2*B*, *red arrows*). This clash is highlighted in the top ranked AF2 model (Fig. S1). Models that are comprised of such energetically unfavorable interactions are unlikely to be useful for downstream applications.

An analysis of the importance of each residue within the epitope for the Nb–peptide interaction was also performed using the AF2 pipeline for Nb_6E_ (Fig. S2). Since there are no experimentally determined structures available for Nb_6E_, we compared the properties predicted for AF2-generated models to those previously acquired in experimental studies (25). After generating a set of AF2 models of Nb_6E_–6E complexes, we evaluated each of these models using a computational Ala scan. ΔΔG values were calculated for each side-chain position within the peptide. As predicted based on visual inspection of the overlaid models, which appeared to show a consistent mode of interaction, variability in ΔΔG values calculated at each position was typically small (Fig. S2). Furthermore, the predicted ΔΔG values showed a qualitative parallel with results from analysis of an Ala-scan library of 6E peptides that was previously published (Fig. S2*B*) (25). Variants of the 6E peptide that showed weak binding in experimental assays mostly possessed Ala mutations at positions that showed high ΔΔG values in the computational Ala scan. One exception is the 6E E5A variant, which showed intermediate binding experimentally and a high ΔΔG value in the computational Ala scan. Conversely, Ala mutations that provided 6E variants that showed strong binding experimentally were typically associated with small ΔΔG values. Further evidence for the accuracy and utility of these models of the Nb_6E_–6E complex relates to the favorable confidence metrics calculated by AF2. The iPTM for all five models of the Nb_6E_–6E complex is ≥0.9, which is similar to parameters calculated for the Nb_Alfa_–Alfa complexes that were shown to be highly accurate (Table S2). These findings show that the AF2 models of the Nb_6E_–tag interaction provide a plausible framework for interpreting and predicting the impacts of structural modifications within the 6E tag.

Prior to this work, no experimental structural data were available for the Nb_127_–127-tag complex, even though it has been used as an epitope tag (32, 38). We experimentally confirmed high affinity binding (*K*_*D*_ – 14 nM) between Nb_127_ and the 127-tag peptide using surface plasmon resonance (SPR) assays (Fig. S3). To provide further context for evaluating the performance of AF2, we crystallized the Nb_127_–127-tag complex (Fig. 3, *A*–*C*) and using X-ray crystallography solved a 2.1 Å structure. The final model was refined to *R*_work_ = 0.18 and *R*_free_ = 0.22, respectively (Table S3). The asymmetric unit contains a single Nb_127_–127-tag complex in a 1:1 binding mode. The model was clearly defined from Glu1 to Ser115 in Nb_127_, with the 127-tag peptide showing a clear fit within the electron density map (Fig. 3*A*). Nb_127_ interacts with the 127-tag *via* five antiparallel beta sheets (β4, β5, β6, β9, and β10) (Fig. 3*B*). This interaction is stabilized by eight strong hydrogen bonds involving residues Glu3′, Phe5′, Trp6′, Lys7′, and Asp10′ of the 127-tag and Tyr37, Lys96, Lys32, Ser54, and Arg52 of Nb_127_. Water-mediated hydrogen bonds also contribute to the interaction, particularly through a network of four water molecules bridging Gly57 of Nb_127_ with Ser1′ and Phe2′ of the 127-tag (Fig. 3*B*). The Nb_127_–127-tag complex is further stabilized by pi–cation interactions between Lys96, Lys32, and Trp6′, as well as pi–pi interactions involving Trp105, Phe2′, and Tyr37, which were observed to reinforce the peptide structure and enhance the engagement between Nb_127_- and 127-tag complex tag (Fig. 3*B*).

We then generated and compared structural models from AF2 for the Nb_127_–127-tag complex to each other and to the experimental data described previously. There was a high degree of structural consistency among the AF2 models (Fig. 3, *C*–*E*), with only a small divergence in the positioning of the C-terminal residues of the 127-tag (Fig. 3*D*, *red arrow*). Overlay and alignment of the experimental structure with the set of AF2 models also reveals a high degree of similarity (Fig. 3*E*). To provide quantitative comparisons of the Nb–tag interfaces seen in AF2 models and in experimental data, we performed computational Ala scans on each of these complexes (Table S4, Fig. 3*F*). There was a high degree of consistency in the predicted energetic contributions (ΔΔG) of 127-tag side chains among the five AF2 models analyzed, resulting in small SDs (Fig. 3*F*). Very similar trends were observed in assessing the experimental Nb_127_–tag complex structure using a computational Ala scan, with the largest divergences observed for the C-terminal residues (positions 9–10, Table S4) in the 127-tag peptide. As for the Nb_Alfa_–tag complex described previously (Fig. 2*A*), we observe a strong positive correlation when plotting ΔΔG values derived from analysis of the experimental structure of the Nb_127_–tag complex *versus* ΔΔG values from analysis of an AF2 model (Fig. 3*F*).This concordance provides further evidence that AF2 can provide models that are accurate and may be useful predicting the impact of peptide epitope structural modifications on Nb binding.

To test whether the AF2 models or the experimental structural data shown above correlated with experimental binding, we synthesized an Ala-scan library of 127 epitope peptides (see Table S5 for sequence information) and assessed their binding to Nb_127_ using a competition-based ELISA (Fig. 3*G*). In this assay, competitor peptides that bind Nb_127_ more strongly block the binding of Nb to immobilized 127 peptide, resulting in higher levels of inhibition. The findings from these experiments parallel findings from computational analyses of AF2 and experimental structural data (Fig. 3, *A*–*C*), which show that important interactions occur between side chains found at positions 2, 3, 5, and 6 within the 127-tag and its Nb binder (Fig. 3*G*, Fig. S4). These similarities provide more support for the utility of AF2 modeling in providing insights into previously structurally uncharacterized Nb–peptide complexes.

To assess whether varying the implementation of AF applied for modeling affected model precision and accuracy, we built and assessed models for each of the Nb–peptide tag pairs using a local version of AF2 (in contrast to the ColabFold running AF2 methodology used above). Assessments of the local AF2-generated models are shown in Table S6. This new assessment shows many similarities when compared with the modeling performed using ColabFold. Two Nb–peptide tag pair models that closely resemble published experimental data (Nb_Alfa_, Nb_PepTag_) are also associated with high AF confidence scores (model_confidence > 0.85, int_plddt > 90). This correlation further supports the hypothesis that a high AF confidence indicates a higher likelihood of structural accuracy. Models for the other two Nb–peptide tag pairs with published crystal structures were annotated with worse assessment scores (“acceptable” and “incorrect” ratings for Nb_Headlock_ and Nb_Moon_ models, respectively). Application of the local AF2 assessment workflow to the two modeled complexes for which there are no previously reported experimental structures (Nb_6E_ and Nb_127_) also resulted in medium-to-high confidence scores, consistency with ColabFold-generated models, and high similarity with newly generated experimental data (Fig. S5). These observations highlight the capability of AF to self-assess the quality of its models to guide further analysis and application of outputs.

A new all-atom version of AF3 reported to improve modeling of Ab–antigen complex structures *versus* AF2 was recently reported (10). We used AF3 to generate models of the six Nb–peptide complexes to benchmark its performance (Table S7). AF3 generated medium or high accuracy top-ranked models for four of five of the structurally characterized complexes, including the Nb_127_–127-tag complex first reported in this work. The AF3 model for the Nb_6E_–6E tag complex agreed with both the AF2 model and experimental findings (Fig. S2). In contrast, AF2 modeling only achieved medium-to-high accuracy in only two-of-five or three-of-five complexes using in-house AF2 or ColabFold analyses, respectively. Although this test set is limited, these results support possible improved performance of AF3 *versus* AF2 for Nb–peptide modeling.

**Figure 4:**
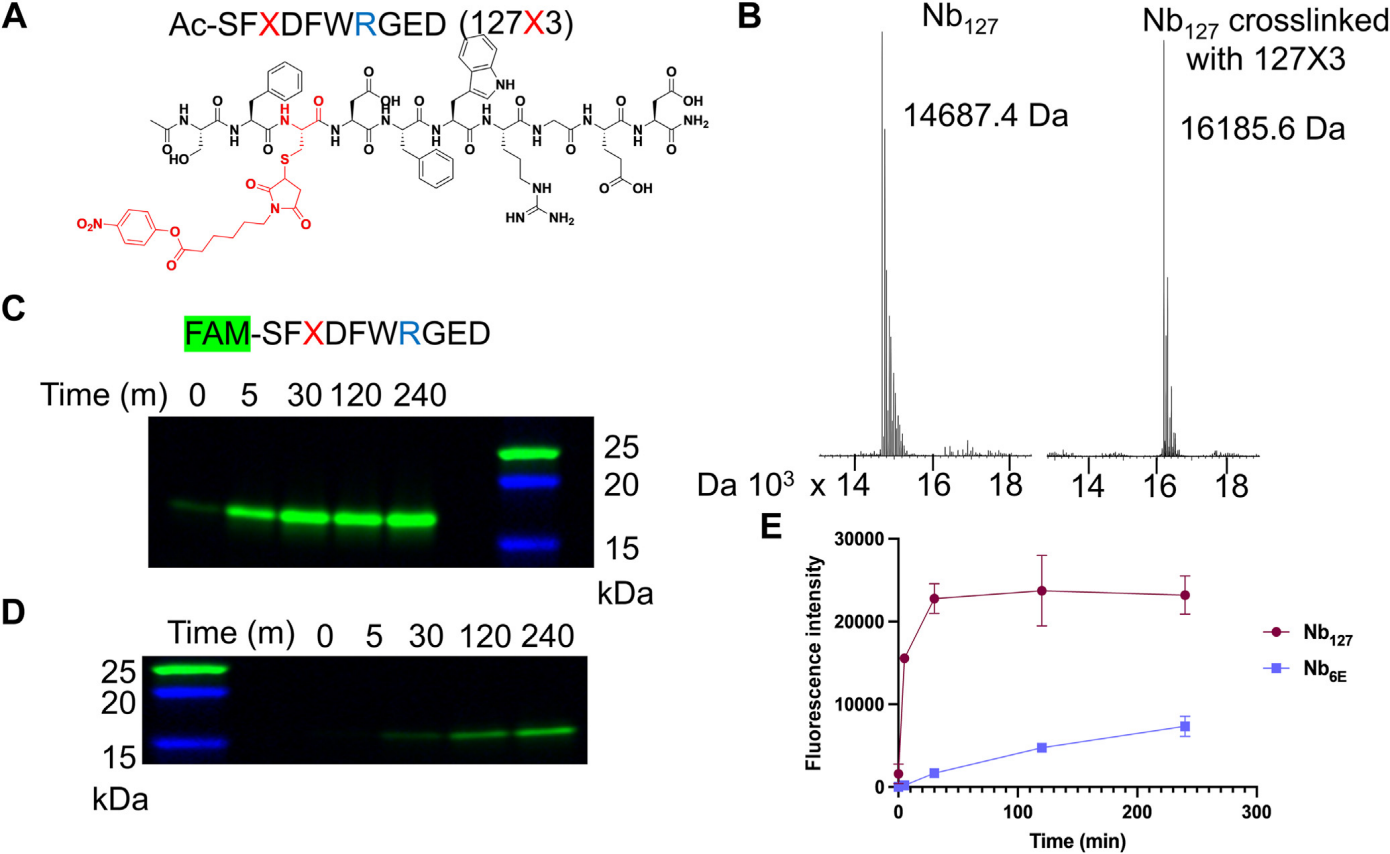
**Evaluation of a crosslinking peptide for Nb_127_ guided by modeling and structure.***A,* structure of an analog of the 127-peptide tag with a crosslinking group (X) incorporated. *B,* mass spectrometry analysis of Nb_127_ before (*left*) and after (*right*) reaction with Ac-127X3. Crosslinking was performed through mixing Nb_127_ (10 μM) and Ac-127X3 (20 μM) in PBS for 2 h at 25 °C followed by purification using a disposable size-exclusion column (PD10). *C,* assessment of the time-dependent formation of a covalently crosslinked complex from Nb_127_ and FAM-127X3 *via* detection of a fluorescent band on SDS-PAGE. *D,* assessment of the reaction of FAM-127X3 and Nb_6E_ (negative control) as described in *C*. Fluorescent gel images were obtained as described in the Experimental procedures section. *E,* quantification of formation of a fluorescent crosslinking product through densitometric analysis of experiments described for *C* and *D*. Fluorescence intensity was quantified using densitometry as described in the Experimental procedures section. The data points are presented as mean ± SD of two independent experiments. An uncropped gel image for data in *C* and *D*, as well as corresponding Coomassie staining, is shown in Fig. S7. Nb, nanobody.

We also asked whether AF2 models or experimental structural data would offer useful insights on the rational design of crosslinking analogs of peptide epitopes. Past work (33) has shown that peptide epitopes linked to electrophiles can rapidly bind and crosslink to a partner Nb, but this approach had not been applied to the Nb–127-tag pair. Peptide-compatible electrophiles used in past studies (phenolic esters) are reported to react with Lys and His residues located near to the interface with binding partners (39). We sought to use the structural data and models described previously to design a peptide–electrophile conjugate that forms a covalent crosslink with its target (Nb_127_). We focused on the incorporation of a crosslinking group at a position within the 127-peptide epitope (Glu3) that was shown (Fig. 3) to form a hydrogen bond with a Lys residue in Nb (Lys96), which provides the requisite functional group for crosslinking. An analog of the 127-epitope peptide containing a Cys at position 3 for crosslinker incorporation and a Lys-to-Arg mutation at position 7 (to avoid autoreactivity) was synthesized using conventional solid phase-peptide synthesis (see Table S5 for characterization). A crosslinking phenolic ester moiety was attached using Cys–maleimide chemistry as previously described (33) to provide a peptide–electrophile conjugate (127X3, Fig. 4*A*). We then tested this conjugate for its ability to form a covalent crosslink with Nb_127_ as evaluated by mass spectrometry (Fig. 4*B*). Mixing Nb_127_ and 127X3 resulted in formation of a new higher molecular weight complex with a mass that corresponded to the formation of the expected crosslinked Nb–peptide product.

We then sought to evaluate the kinetics and specificity of crosslinking between Nb_127_ and 127X3. To facilitate easier tracking, we synthesized an analog of 127X3 containing a carboxyfluorescein fluorophore at its N terminus (FAM-127X3, Table S5). We evaluated the kinetics of crosslinking between FAM-127X3 and Nb_127_ or Nb_6E_ as a negative control (Fig. 4, *C*–*E*). The mixture of FAM-127X3 and Nb_127_ forms a band corresponding to the crosslinked Nb–peptide product within 5 m, whereas the corresponding reaction with Nb_6E_ is much slower, with a fluorescent band only appearing after 60 m (Fig. 4, *C* and *D*). Addition of a noncrosslinking competitor peptide with high affinity for Nb_127_ (127A7) completely blocked formation of the fluorescently labeled Nb–peptide crosslink product (Fig. S6). The difference in the kinetics of labeling Nb_127_ and Nb_6E_ with FAM-127X3 was quantified through densitometric measurement of the fluorescent band intensity (Fig. 4*E*). This comparison confirms that the crosslinking performance of 127X3 is accelerated through binding Nb_127_. Despite the rapid initial crosslinking, a qualitative analysis of crosslinking conversion by Coomassie staining shows that crosslinking does not proceed to full conversion (Fig. S7). The cause of this stall in crosslinking conversion is unknown and will be investigated in future mechanistic studies.

**Figure 5:**
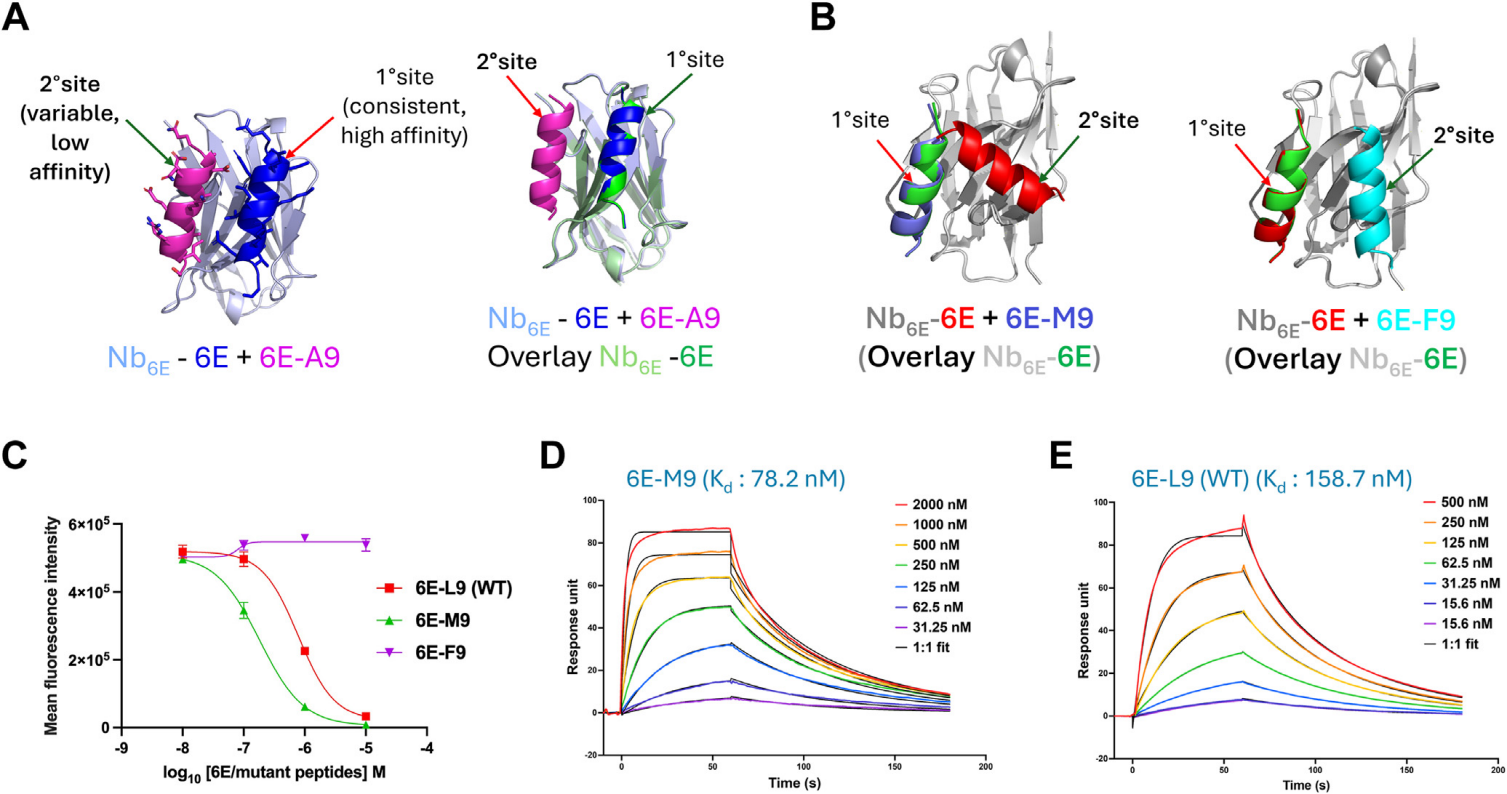
**Competition binding using AF2 parallels experimental findings.***A, left,* illustration of primary and secondary binding sites on Nb_6E_ for epitope variants with differing affinities. *Right,* illustration of retention of primary binding site for high-affinity epitope (6E) when in competition with a lower affinity variant peptide (6E-A9). *B,* three component binding models from AF2 predict that one variant peptide (6E-M9, *left*) outcompetes 6E for binding to the 1° binding site on Nb_6E,_ whereas a different variant peptide (6E-F9, *right*) does not. *C,* experimental flow cytometry competition binding assays to assess 6E variant peptide binding. HEK293 cells stably expressing a cell surface protein fused with Nb_6E_ (A2AR-Nb6E-ALFA) were coincubated with variable concentrations of variant 6E peptides (10–0.01 μM) and a fixed concentration (10 nM) of fluorescein-labeled 6E peptide (FAM-6E-C14), followed by washing, and detection with antifluorescein antibody conjugated with AlexaFluor647 (AF647) (see the Experimental procedures section). Binding was quantified as median AF647 fluorescence intensity (see Fig. S10 for representative histograms). *D* and *E,* representative SPR sensorgrams showing association and dissociation curves for interaction of 6E-M9 (2–0.03 μM) and 6E-L9 (WT) (0.5–0.02 μM) with Nb_6E_-biotin immobilized on a streptavidin (SA) chip (Cytiva; catalog no.: 29104992). Experimental details are described in the Experimental procedures section. AF2, AlphaFold2; HEK293, human embryonic kidney 293 cell line; SPR, surface plasmon resonance.

We also sought to assess whether AF2 modeling could discern between analogs of peptide epitopes that had been shown to bind to Nbs of interest with differing affinity, with analogy to a previously described competition binding approach (40). A three-component input consisting of Nb_6E_, 6E–peptide, and 6E–A9 peptide was used to generate a set of models. Past experimental work had shown that Nb_6E_ bound 6E with substantially higher affinity than the 6E-A9 variant (25). The models generated by AF2 from the three-component input consistently showed 6E peptide bound at a primary site (1°, Fig. 5*A*) that overlays with the site occupied by 6E peptide in models generated from a two-component (Nb_6E_ and 6E) input. In contrast, these three-component models showed 6E-A9 placed in contact with Nb_6E_ at a secondary (2°) binding site that was more variable between models (Fig. S8). AF2 generated assessments of confidence in localized protein structures (predicted local distance difference test [pLDDT]) also reveal differences between the peptide that binds at the 1° binding site (6E) and the weaker binding peptide (6EA9, Fig. S8).

We then assessed whether AF2 could be used to predict the relative affinity of novel 6E analogs for Nb_6E_ to prioritize subsequent experimental studies. First, 6E peptide variants containing modifications at a site (position 9, Leu in WT 6E peptide) that were sensitive to an alanine substitution were evaluated using AF2 (25). An input consisting of Nb_6E_, 6E-L9 (WT), and 6E-M9 showed that the 6E-M9 variant peptide consistently outcompeted 6E-WT for binding to the primary site on Nb_6E_ (Fig. 5*B*, Fig. S9). In contrast, 6E-F9 was uniformly outcompeted by 6E-WT. These predictions were then tested experimentally. The binding affinity of peptides for Nb_6E_ was quantified using a competition binding assay (Fig. 5*C*, Fig. S10) (25) and with SPR (Fig. 5*D*). The results from both the assays are in agreement and confirm AF2 predictions; the rank of binding affinities in each assay is: 6E-M9 > 6E-WT > 6E-F9. This finding suggests that future studies to enhance the affinity of 6E for Nb_6E_ may benefit from the use of flexible alkyl or heteroalkyl chains at position 9, while aromatic side chains should be avoided.

We also assessed whether the incorporation of a residue with a large aromatic side chain (tryptophan) would be tolerated at other positions within the 6E peptide that were amenable to Ala substitution (Fig. S2). Both AF2 and SPR analyses show that these peptides (6E-W4, 6E-W7, and 6E-W8) bind Nb_6E_ with affinity that is improved relative to 6E-WT (Fig. S11). In the course of these assessments, we also compared 6E-WT to an analog with three glycines appended at the N terminus (G_3_-6E), used widely in previous studies (25, 33). Surprisingly, G_3_-6E was superior to 6E-WT both in competition binding assays and as assessed by SPR (Fig. S12). A comparison of 6E-WT *versus* G_3_-6E binding to Nb_6E_ by AF2 did not match experimental observations, perhaps related to difficulties in modeling and assessing disordered peptide termini (Fig. S12) (41).

**Figure 6:**
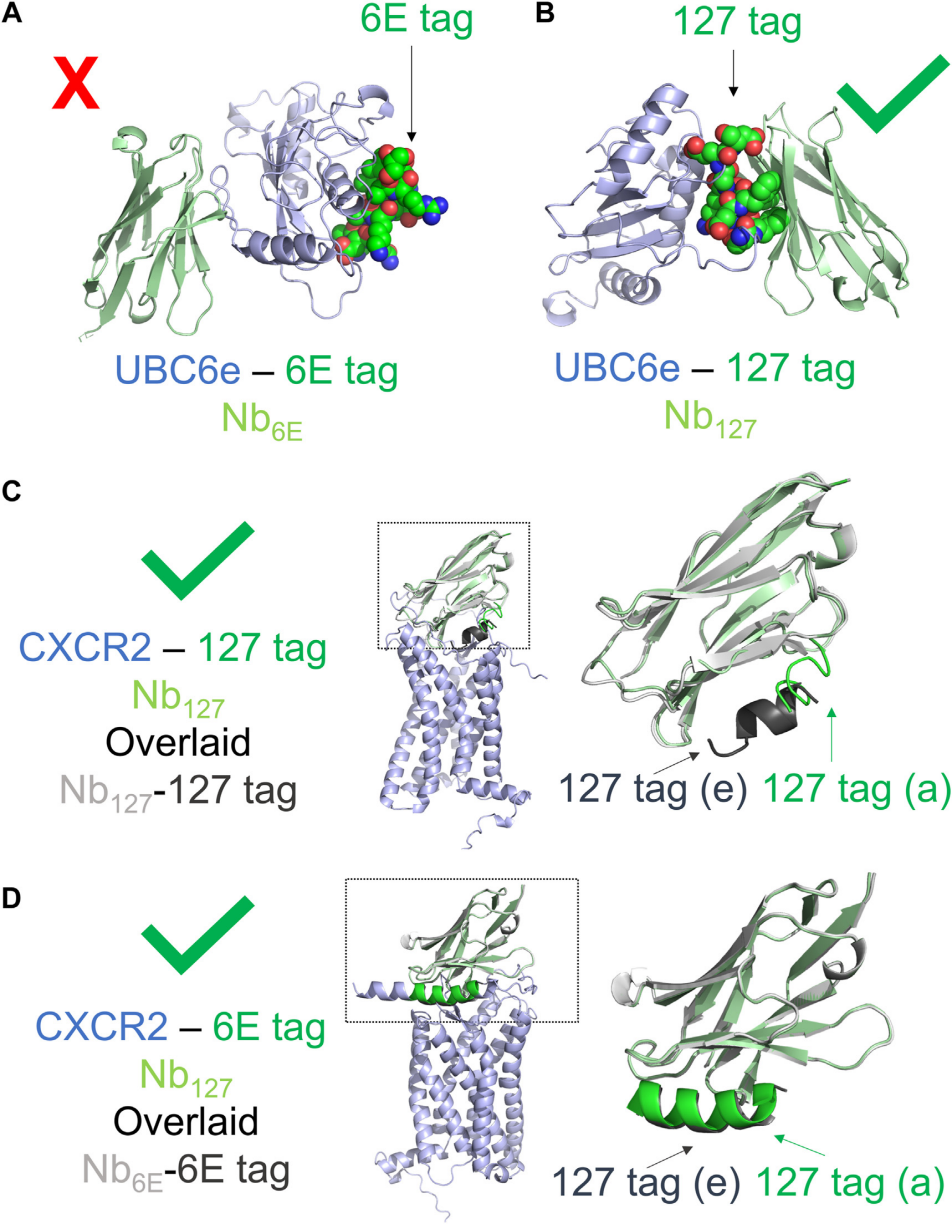
**Evaluation of AF2 modeling using full-sized Nb antigens.***A,* AF2 fails to predict experimentally observed binding of Nb_6E_ (*light green*) to 6E epitope (*green spheres*, *right*) when found in the context of UBC6e antigen (*light blue*). *B,* AF2 successfully predicts binding of Nb_127_ (*light green*) binding to 127-tag epitope (*green spheres*) when found in the context of UBC6e antigen (*light blue*). *C,* AF2 modeling of Nb_127_ (*light green*) binding to 127-tag epitope (*green*) when found in the context of full-sized CXCR2 antigen (*light blue*) shows acceptable overlap with the AF2 model of Nb_127_–127-tag (*light gray*, *dark gray*) alone. *D,* AF2 modeling of Nb_6E_ (*light green*) binding to 6E-tag epitope (*green*) when found in the context of full-sized CXCR2 antigen (*light blue*) shows good overlap with the AF2 model of Nb_6E_-6E tag (*light gray*, *dark gray*) alone. *Insets* from *dashed boxes* (*C* and *D*) are shown at *right*. The label “(e)” refers to modeling of the peptide as an epitope, and the label “(a)” refers to modeling of the peptide within the full-size antigen. All alignments are performed using PyMOL as described in the Experimental procedures section. AF2, AlphaFold2; Nb, nanobody.

The effective modeling of selected Nb–peptide epitope interactions led us to probe what role the protein context had on the success of modeling. We thus assessed whether Nb–peptide epitope complexes modeled successfully in isolation would also be modeled appropriately in the context of full-size antigen–Nb complexes. Recently published assessments of using AF to identify Nb epitopes demonstrated accuracy at a modest level (∼50–70%) (42). Previously published experimental data show that both Nb_6E_ and Nb_127_ bind the sequences used in epitope tag development when found in the context of intact protein (19, 24, 32, 38). We thus generated models of Nb_6E_ with intact antigen (Fig. 6*A*, UBC6e-6E tag), Nb_127_ with UBC6e modified to replace the 6E tag with 127-tag (Fig. 6*B*, UBC6e-127-tag), Nb_127_ with intact antigen (Fig. 6*C*, CXCR2-127-tag), or Nb_6E_ with CXCR2 modified to replace the 127-tag with the 6E tag (Fig. 6*D*, CXCR2-6E tag). Models of the UBC6e–Nb_6E_ complex were not consistent with past experimental data (24), with Nb_6E_ binding at the face of UBC6e opposite the 6E tag, with substantial variability between models (Figs. 6*A*, S13). This observation was surprising, given the good agreement between AF2-generated models of the Nb_6E_–6E tag complex and experimental findings characterizing this interaction (Fig. S2). Complications introduced by modeling a complex with full-size UBC6e instead of the isolated tag are likely to be the source of this loss in performance. Notably, the fragment of UBC6e that corresponds to the 6E tag associates with the UBC6e core and buries residues important for binding Nb_6E_ when modeled in this context (Fig. S14). Performing an analogous modeling between Nb_127_ and UBC6e-127-tag provided self-consistent models in which the Nb associated with tag, even in an unnatural context (Fig. 6*B*). Modeling the interaction of Nb_127_ with CXCR2-127-tag, the antigen used for immunizations to generate Nb_127_ (43), showed an acceptable concordance with the models of the Nb_127_–127-tag complex in isolation (Fig. 6*C*, *right*), in line with the effective use of the Nb_127_–tag pair in a variety of biological contexts (32). Finally, modeling of Nb_6E_ with CXCR2-6E tag also provided a complex that closely overlaid with the Nb_6E_–6E tag complex in isolation (Fig. 6*D*). Overall, AF2 generated self-consistent models in three of four test cases, demonstrating its utility for offering Nb–protein complex models to serve as a starting point for hypothesis generation.

## Discussion

The release of AF2 and subsequent iterations has furthered scientists' capability to predict the structure of proteins and protein complexes (5). Its adaptation has been empowered by the development of tools, such as the ColabFold platform (34), that allow users without extensive modeling expertise to generate models that lead to new hypotheses and research directions. Despite widespread adaptation, one of the least successful areas for AF prediction (and predictions of PPIs more generally) relates to its modeling of Ab–antigen structures. The causes for this challenge are multifaceted; intense and ongoing studies are underway to improve performance in this area (7, 8, 9). In this work, we perform a focused assessment of the performance of AF2 for generating useful and accurate models of Nbs bound to their targets. Previously published, broad assessments of AF2 performance have evaluated its performance for some Nb–antigen complexes (8, 42). Here, we have delved into this analysis, structurally characterized a novel Nb–epitope interaction, and have tested the utility of AF2 models for creating crosslinking Nb–peptide epitope pairs.

There are several previous studies that have focused on the application of AF for modeling the binding of small peptides to full-sized protein targets (40, 44, 45, 46, 47, 48) as well as the binding of Abs to antigens (8, 9, 42, 49). The findings in this work generally align with results from past studies. In one example, AF2 has been used to model the binding of small peptides to G protein–coupled receptors (46). Recent work in the CAPRI initiative has provided insights into the performance of AF2 for modeling Ab–peptide (and Ab–protein antigen) interactions, showing that strategies such as additional AF2 sampling can be helpful, while accurate scoring and model selection is an ongoing challenge (50, 51, 52). Initially, the success of AF2 in modeling such interactions was surprising, given that most of these small peptides do not adopt a folded structure in solution. However, peptide binding and folding often occur together. As such, these interactions are analogous to interactions between distant sequences within individual proteins, which are effectively modeled by AF2. In this context, it is interesting to note that most of the Nb–tag pairs modeled effectively (Alfa, 127, 6E, and PepTag) are comprised of a peptide epitope that adopts (at least partially) a regular secondary structure (alpha helix). This alone does not guarantee success, as another Nb-bound epitope peptide (Moon) adopts a partially helical conformation upon binding but is modeled incorrectly. We also found that the protein context in which the epitope is situated affects modeling success. Modeling of Nb_6E_ with its 6E peptide epitope alone provides a model consistent with experimentally observed trends. In contrast, modeling Nb_6E_ with 6E epitope found in the context of full-sized antigen (UBC6e) generates models inconsistent with experimental findings. This divergence resulting from variation in modeling inputs has not been widely noted in previous studies. Future efforts to model Ab–antigen (or protein–peptide) interactions may benefit from comparing models produced using full-sized antigens to those generated with isolated epitopes, when possible.

In this work, we find AF2 to be modestly successful in generating accurate and useful models of Nb–peptide epitope complexes. For four of six examples, AF2 generates models that either closely mimic experimentally derived structural data or are in good concordance with experimental binding data (Tables S1 and S5). For the other examples, AF2 produces models that are not internally consistent and do not conform with experimental data. In this context, it is worth noting that both Nb–tag pairs that were poorly modeled by AF2 were included in the training set. Memorization of the training set by AF2 is thus no guarantee that models of complexes included in the training will be accurate. A newer version of AF (AF3) showed improved performance for modeling some complexes (compare Tables S6 and S7). Recent studies have shown that even when AF2 models show modest divergences with experimental structural data, they can still be useful for docking campaigns to identify new binders (53). It is possible that the AF2 models generated in this work that diverge from experimental structures could still be useful, although further studies will be required to assess this possibility. The determinants of success for generating models of Nb–peptide tag interactions that resemble experimental findings remain unclear. One potentially noteworthy observation relates to a correlation between the length of Nb CDR3 loops, which correspond to the most diverse portion of Nbs sequences, and modeling success. The two Nb–tag pairs modeled least successfully (Nb_Moon_ and Nb_Headlock_) are comprised of Nbs with the longest CDR3 regions (14 and 13 residues, respectively). However, it is not possible to make any firm conclusions on the relationship between CDR3 length and modeling success from this small dataset. In summary, these findings demonstrate that AF2 modeling can generate precise and accurate models of Nb–tag interactions, which can be leveraged to design new analogs of epitopes with useful properties such as covalent crosslinking capacity.

## Experimental procedures

### Modeling protein–protein complexes using AF

Modeling was performed with AF2 using the online tool ColabFold (v1.5.5) (34) as well as the full downloaded AF2 pipeline. For ColabFold, the Alphafold2_multimer_v3 model was applied for prediction of protein complex structures. Following default ColabFold AF2 settings, output models were not subjected to relaxation, and no structural templates were used. The MSA mode was mmseqs2_uniref_env. The “unpaired_paired” mode was used in which sequences from the same species and unpaired MSA were paired. A maximum of three recycles were allowed along with the “auto” setting for early stop recycle tolerance. The pairing strategy was set to the “greedy” setting. The “max_msa” setting was placed on “auto,” and the number of seeds set to “1.” Bipartite and tripartite inputs were submitted with “:” separating each separate polypeptide chain.

The full AF2 pipeline was downloaded from GitHub and installed on a local computer cluster and run with the AF-Multimer v2.2 model. To avoid overlap of templates with modeling targets, a template date cutoff of April 6, 2016 was used for the Nb_PepTag_ and Nb_Headlock_ complexes (corresponding to the Nb_Headlock_ complex, Protein Data Bank [PDB] code: 5IVN, release date), whereas a template date cutoff of April 30, 2018 was used for the remaining complexes. Twenty-five models were generated per complex, and the top-ranked model based on AF2 confidence score was assessed. Models were relaxed using the AF2 Amber relax protocol. Model confidence metrics pTM, ipTM, and confidence score were obtained from AF2 output, and interface pLDDT was calculated from output residue pLDDT scores from modeled structures, averaging all pLDDT values for residues within 4 Å of the binding partner.

The AF3 pipeline was run on a local computer cluster, with template structures excluded, both to avoid overlap with modeling target structures and for consistency with the tested AF2 protocols, which used no templates (ColabFold) or restricted templates (downloaded AF2). Twenty-five modeled structures were generated per complex (five seeds), and the top-ranked model based on AF3 ranking score was assessed. As with AF2, AF3 confidence scores were obtained directly from output (pTM, ipTM, and ranking score) or calculated from interface residue confidence scores in modeled structures (interface pLDDT). As pLDDT scores in AF3 vary between atoms in a given residue, residue pLDDT scores were obtained by averaging all atom pLDDT values for each residue.

### Generation of input sequences for AF modeling

Nb, epitope tag, and protein sequences were extracted either from the UniProt database, the PDB, or from published sequences. Full sequences for each Nb and epitope tag used in modeling experiments are found in Supporting information. Modeling experiments using full-length proteins were truncated as listed either in figure captions or in Supporting information. Chimeric sequences in which epitope tags are swapped into heterologous full-length proteins are also listed in Supporting information.

### Structure model visualization using PyMOL

Structure models generated by AF2 were visualized using PyMOL (version 2.4.1). Alignments of AF2 outputs were performed using the “Align all to this” command. Visualizations were created showing cartoon, surface, or mesh depictions as described in figure captions.

### Model accuracy assessment

Model accuracy metrics were computed based on comparison of modeled complexes with the corresponding X-ray structures using the DockQ program (54). Calculated metrics include interface RMSD, corresponding to backbone RMSD of modeled *versus* X-ray structure residues at the Nb–peptide interface, peptide RMSD, corresponding to backbone RMSD of modeled peptide *versus* X-ray structure peptide after superposition of the Nb, and CAPRI accuracy level (35). CAPRI model accuracy level is based on a combination of interface RMSD, ligand RMSD (peptide RMSD in this context), and fraction of native contacts in the modeled complex and classifies each model into one of four accuracy levels: incorrect, acceptable, medium, and high. The protein–peptide complex CAPRI criteria were used for assessment of these complexes (“-capri_peptide” flag in DockQ) (35).

### Computational alanine scan to assess contributions of peptide tag side chains to binding

PDB files generated either as AF2 outputs or retrieved from the PDB were used as inputs for into the online BUDE Alanine Scan tool (36, 37). Experimental data were processed using PyMOL to remove water molecules prior to input into the online tool, but no other preprocessing was performed. Output ΔΔG values were used to generate graphics and tables shown in figures as described in legends.

### ELISA analysis of Nb_127_ binding by 127-tag peptide analogs

Immobilization of 127-tag peptide was performed by addition and overnight incubation of 127-tag peptide conjugated to green fluorescent protein (using sortagging, see later) on Nunc Maxisorp 96-well plates at a dose of 100 ng/well. Wells were then washed with PBS with 0.05% (v/v) Tween-20 (PBST). Wells were then treated with a mixture of Nb_127_–biotin (60 nM, prepared using sortagging) and competitor peptides (concentration 100 μM) for 1 h. Following incubation, all wells were washed 3x with PBST, and streptavidin–horseradish peroxidase (BioLegend; #405210; 1:2000 dilution) was added for 30 min. Washing with PBST (3x) was performed, and the plate was treated with TMB ELISA solution (Thermo Fisher; #34028). Development was quenched by addition of 1 M sulfuric acid, and absorbance was recorded at 480 nm.

### Protein labeling *via* sortagging

Sortagging reactions were performed using Sortase 5M to label target proteins at their C terminus as previously described (19). Reactions were purified with nickel–nitrilotriacetic acid affinity chromatography and size-exclusion chromatography (PD10 columns; Cytiva, #17085101) to yield site-specifically labeled proteins.

### Protein expression and purification

Nb protein sequences from the literature (see Supporting information) were codon optimized for bacterial expression and cloned into a pET26b expression in frame with pelB and His6 sequences using clone EZ service from GenScript. The production and purification of Nbs has been described previously (19). The identity of the purified Nbs was confirmed by mass spectrometry, and the concentration of Nbs was determined by measuring the absorbance at 280 nm.

### Mass spectrometry characterization of purified peptides and proteins

Mass spectrometry data were acquired on a Waters Xevo qTOF LC/MS instrument in positive ion mode. For proteins analyzed by mass spectrometry, singly charged ions were not observed, so protein intact mass was calculated from analysis of multiply charged ions using the MaxENT algorithm on MassLynx (Waters) software.

### Peptide synthesis, purification, and modification with a crosslinking group

Alanine scan peptide analogs of 127-tag were ordered *via* custom synthesis from GenScript and purified before further use (see later). All peptides used for construction of cross-linking conjugates were synthesized in house *via* solid phase peptide synthesis with Fmoc protection of the amine backbone on a Gyros PurePep Chorus or Liberty Blue Microwave-Assisted Automated Peptide Synthesizer. Peptide synthesis was performed on Rink Amide resin to afford a C-terminal carboxamide. Fmoc-amino acids were dissolved in dimethylformamide (DMF) and added to resin with Oxyma and diisopropylcarbodiimide. Fmoc groups were deprotected using 10% to 20% piperidine in DMF. For crosslinking peptides, the N terminus was acetylated through treatment of washed resin with a mixture of acetic anhydride, diisopropylethylamine, and DMF (1:2:8 v/v) for 10 m.

Synthesized peptides were cleaved from the resin using a cleavage cocktail comprised of TFA/H_2_O/triisopropylsilane (92.5:5:2.5% by volume) and rocked at room temperature for 3 h prior to filtration. Crude peptides were precipitated using diethyl ether. Peptides were purified *via* preparative-scale HPLC using a Phenomenex Aeris Peptide XB-C18 Prep column (particle size, 5 μM; pore size, 100 Å) with a linear gradient of solvent A (0.1% TFA in H_2_O) and solvent B (0.1% TFA in acetonitrile). Fractions containing peptides of interest were identified using mass spectrometry analysis. Mass spectrometry characterization of peptides is shown in Table S5.

Fluorescein was appended to the peptide N terminus on resin. To the solid support containing crude, protected peptide, 10 equivalents of 5(6)-carboxyfluorescein (Acros Organics), 10 equivalents of hexafluorophosphate azabenzotriazole tetramethyl uranium, and 20 equivalents of diisopropylethylamine were added. The reaction was shaken overnight and washed with DMF. Peptide cleavage and purification was performed as described previously.

Peptide electrophile (p-nitrophenol ester) conjugates were synthesized as previously described (33). Briefly, purified peptides containing Cys residues were dissolved in dimethyl sulfoxide (10 mM) and mixed with three to five equivalents of maleimide–phenol ester, which was produced according to a published protocol (33). Concentrated phosphate buffer (1 M, pH 7.5) was then added (final concentration of 100 mM). The reaction was shaken at room temperature for 1 h, and the product was purified by reverse-phase HPLC (C18 column, gradient 20–90% acetonitrile in water with 0.1% trifluoroacetic acid), lyophilized, and dissolved in dimethyl sulfoxide (1 mM stock) prior to use. Conjugate identity was confirmed *via* mass spectrometry (Table S5).

### Peptide crosslinking and assessment by SDS-PAGE

Crosslinking reactions were performed using one equivalent of Nb (10 μM) and two equivalents of 127X3 crosslinking peptide (20 μM) in PBS at 25 °C for 2 h. The reaction mixture was then purified using fractionation with a size-exclusion PD-10 column (Cytiva; #17085101). Fractions containing Nb were concentrated using a 10 KDa molecular weight cutoff centrifugal filter (Sigma–Aldrich; UFC9010). Reaction progress was monitored by SDS-PAGE and mass spectrometry.

The reaction of Nb_127_ and FAM-127X3 was performed as described previously, with the following modifications. The reaction was monitored over 4 h. Aliquots were collected at different time points (0, 5, 30, 120, and 240 m) and quenched using a solution of 100 mM DTT in 4x Laemmli sample buffer (Bio-Rad; catalog no.: 1610747) followed by heating at 95 °C for 10 m. Denatured samples were resolved using electrophoresis on a 4% to 20% acrylamide gradient SDS-PAGE gel (Bio-Rad; catalog no.: 4561093). The gel was first scanned to detect fluorescence signals, followed by total protein staining using Coomassie stain (Protein Gel Stain; Biotium, catalog no.: 21003). Fluorescence scans were obtained using the Flamingo (590/110; Blue Epi/UV filter) application and merged with a Coomassie Blue Far Red Epi application (715/30 Filter) in the ChemiDoc MP system (Bio-Rad).

Images were processed for quantification using FiJi software (ImageJ) and by selecting an equal rectangular area for all the fluorescent bands. The mean and SD for the integrated signal for each band were calculated from two independent experiments.

### X-ray crystallography

A fresh preparation of Nb was expressed and purified prior to crystallization. The Nb (5 mg/ml) was mixed with its corresponding peptide epitope tag at molar ratios of 1:2 and 1:3, respectively, at 4 °C overnight. The mixture was then centrifuged at high speed to remove debris and any unbound Nb. The crystallization screening was performed using an Art Robbins Gryphon crystallization robot by hanging-drop vapor diffusion method. The screening was performed in a 96-well format (192 conditions) using the Index HT (Hampton Research; #HRT-144) crystallization kit. Nb–peptide (1 μl) was mixed with 1 μl reservoir solution and then equilibrated against 50 μl reservoir solution, and plates were stored at 291 K. Crystal drops were observed daily using a bright-field microscopy. Diffraction-quality crystals of the Nb_127_–127-tag complex were obtained in condition containing 0.1 M Tris (pH 8.5) and 2.0 M ammonium sulfate. Diamond-shaped crystals appeared after 3 days of equilibration against the crystallization solution and grew to full size of 0.3 × 0.4 × 0.5 mm in 7 days.

### Data collection and structure determination

For data collection, the crystals were briefly soaked in reservoir solution supplemented with 25% (v/v) ethylene glycol and then flash-cooled in liquid nitrogen. X-ray diffraction data were collected at the National Institute of Diabetes and Digestive and Kidney Diseases Molecular Structure Facility using a Rigaku Raxis-IV+ and EIGER 4M pixel array detector. Diffraction data were processed using the HKL2000 package (55). The crystal structure described in this study was solved by molecular replacement using the model of PDB accession code 6B20 (chain E) as a search model and model building used AutoBuild in Phenix1-1.20.1 (56). After several cycles of manual adjustments with Coot 1.0 (57), the model was refined with Phenix giving a final *R* and *R*_free_ of 0.18 and 0.22, respectively; structural statistics are reported in Table S3. All structure figures were generated using PyMOL 2.3.2 (The PyMOL Molecular Graphics System; Schrodinger). The coordinates and structure factors have been deposited in the PDB with accession code 9NK9.

### Flow cytometry analysis of peptide binding to human embryonic kidney 293 cells

Human embryonic kidney 293 cells stably expressing a cell surface protein fused with Nb_6E_ (A2AR-Nb_6E_-ALFA, described previously (25)) were cultured in Dulbecco's modified Eagle's media containing 10% fetal bovine serum (Thermo; #A5256801) and 1X penicillin–streptomycin (Thermo; #15140-122). Binding assays were performed as described previously (25). Briefly, cells were suspended in 2% bovine serum albumin (BSA) in 1X PBS (w/v) (BSA-PBS) with variable concentrations of unlabeled variant 6E peptides (10–0.01 μM) and 10 nM fluorescein-labeled 6E peptide (FAM-6E-C14) for 30 m followed by 2x washing with BSA-PBS, and subsequent staining with antifluorescein Ab conjugated with AlexaFluor647 (1.5:1000 dilution in BSA-PBS; Jackson ImmunoResearch; #200-602-037). Cells were incubated again for 30 m, pelleted, washed, and analyzed by flow cytometry on a CytoFlex flow cytometer (Beckman Coulter). Cell labeling intensity was monitored in the FL4-APC channel. Live cells were identified and gated based on forward scatter–side scatter profile. A set of 2000 events corresponding to live cells were captured. Median fluorescence intensity for cell counts observed in the APC channel for each sample was calculated from histograms. Median fluorescence intensity values obtained from technical replicates of the same sample were averaged and plotted against the corresponding concentration of the variant 6E peptide.

### SPR analysis for Nb–tag binding affinity

SPR biosensor analysis was conducted on a Biacore T200 instrument (Cytiva) using a Series S Sensor Chip SA (streptavidin; Cytiva, #29104992) to determine Nb–tag binding affinity. All binding analyses were done in degassed SPR running buffer (1X PBS at pH 7.4 plus 0.05% P20). Biotinylated Nbs (Nb_6E_ or Nb_127_), produced by sortagging reaction, was captured on SA chip where streptavidin is covalently coupled to dextran matrix. Biotinylated Nb (1 μM) was immobilized on flow cell *via* biotin–streptavidin interaction to reach a target response value of approximately 2000 response units. A separate flow cell was capped with only SPR running buffer to use as a surface control for nonspecific binding.

Analyte samples (127-tag peptide or mutant-6E peptides) were prepared as serial dilutions in SPR running buffer. Analyte was flowed over the chip at 50 μl/min with a contact time of 60 s and a dissociation time of 120 s. A duplicate injection of one concentration and a buffer injection (blank) were used for assessing chip surface performance and double referencing, respectively. The regeneration step (dissociation of bound analyte) included consecutive washes with 10 mM glycine solution (pH 1.5), followed by running buffer for reconditioning. Kinetic constants and *K*_*D*_ values (M) were calculated from local fittings using 1:1 kinetics binding model for peptide (analyte) binding to Nb–biotin on the BIAevaluation software (Cytiva).

## Data availability

All data describing the results of this article are available either within the article, the supporting information, or in the PDB entry described previously.

## Supporting information

This article contains supporting information (54).

## Conflict of interest

The authors declare that they have no conflicts of interest with the contents of this article.
